# Short and long-term outcomes of surgical intervention for empyema in the post-fibrinolytic era

**DOI:** 10.1186/s13019-021-01566-z

**Published:** 2021-07-02

**Authors:** Caitlin J. Cain, Marc Margolis, John F. Lazar, Hayley Henderson, Margaret Hamm, Stefanie Malouf, Puja Gaur Khaitan

**Affiliations:** 1 Department of General Surgery, Georgetown University School of Medicine, Medstar Washington Hospital Center, 110 Irving Street, NW (G253), Washington DC, 20010 USA; 2 Division of Thoracic Surgery, Georgetown University School of Medicine, Medstar Washington Hospital Center, 110 Irving Street, NW (G253), Washington DC, 20010 USA

**Keywords:** Empyema, Open window Thoracostomy, Bronchopleural fistula

## Abstract

**Background:**

Open window thoracostomy (OWT) is indicated for patients with bronchopleural fistula (BPF) or trapped lung in the setting of empyema refractory to non-surgical interventions. We investigated the role of OWT in the era of minimally invasive surgeries, endobronchial valves and fibrinolytic therapy.

**Methods:**

A retrospective chart review of all patients who underwent OWT at a single institution from 2010 to 2020 was performed. Indications for the procedure as well as operative details and morbidity and mortality were evaluated to determine patient outcomes for OWT.

**Results:**

Eighteen patients were identified for the study. The most common indication for OWT was post-resectional BPF (*n* = 9). Prior to OWT, *n* = 11 patients failed other surgical or minimally invasive interventions. Patient comorbidities were quantified with the Charlson Comorbidity index (*n* = 11 score ≥ 5, 10-year survival ≤21%). Three (16.7%) patients died < 30 days post-operatively and 12 (66%) patients were deceased by the study’s end (overall survival 24.0 ± 32.2 months). Mean number of ribs resected were 2.5 ± 1.2 (range 1–6) with one patient having 6 ribs removed. Patients were managed with negative pressure wound therapy (*n* = 9) or Kerlix packing (*n* = 9). Eleven patients (61.6%) underwent delayed closure (mean time from index surgery to closure 4.8 ± 6.7 months).

**Conclusions:**

Our study illustrates the significant comorbidities of patients undergoing OWT, the poor outcomes therein, and pitfalls associated with this procedure. We show that negative pressure wound therapy can be utilized as potential way to obliterate the pleural space and manage an open chest in the absence of an airleak; however, OWT procedures continue to be extremely morbid.

## Background

Since the inception of pleural fibrinolytic therapy in 2011 for management of empyema and complicated parapneumonic effusions, the incidence of decortication has substantially declined [1–4]. Surgical management is considered for empyema when non-surgical therapy – i.e. antibiotics, tube thoracostomy, fibrinolytics -- fails as well as in cases presenting as organized empyema with lung entrapment [5, 6]. Surgical interventions include video assisted thoracoscopic surgery (VATS) decortication and open thoracotomy with decortication. For unstable patients, Clagett type open-window thoracostomy (OWT) with rib resection and modified Eloesser flap (MEF) with rib resection both with or without negative pressure wound therapy (NPWT) are options, as both operations allow for continuous access to the pleural space for pus evacuation and debridement [6–10].

**Fig. 1:**
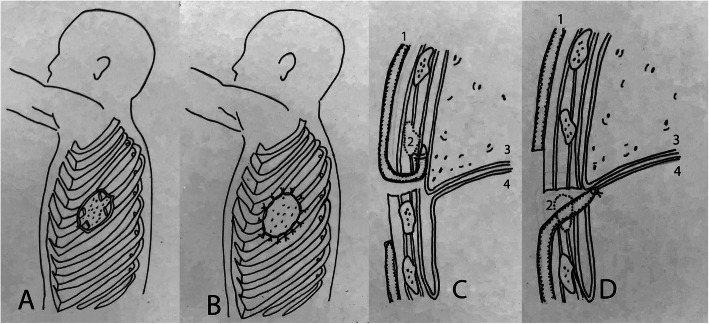
**A** OWT part I with rib segments removed showing underlying lung parenchyma; **B** OWT part II showing skin sutured circumferentially to parietal pleura (marsupialization). **C** Eloesser flap (adapted from original sketch by Dr. Eloesser) [11], **D** Modified Eloesser flap with numbers 1, 2, 3, 4 corresponding to cutaneous flap, removed rib segment, lung parenchyma, and diaphragm respectively

An OWT is a procedure which may include elements of the Clagett window, Eloesser Flap (EF), or MEF, but fundamentally involves removal of one or more rib segments and circumferentially marsupializing the parietal pleura to the skin (Fig. 1A,B). In 1935, Eloesser described the original procedure for open chest drainage, with creation of a U-shaped cutaneous flap sutured apically under a resected rib segment (Fig. 1C) [11]. In 1963, Clagett and Geraci described a new drainage procedure which entailed the removal of one rib segment and suturing the superficial fascia down to the periosteum of the resected rib [7]. The MEF was introduced in 1971 by Symbas et al. with the idea of providing superior empyema decontamination by a dependent drainage system not present in the traditional EF. The MEF is an inverted U-shaped cutaneous flap sutured inferiorly to the diaphragm below a segment of resected rib (Fig. 1D) [12]. In contrast to the traditional Clagett window and OWT, where the ultimate goal is chest closure after successful empyema treatment, EF and MEFs are historically closed primarily through epithelialization and obliteration of the empyema cavity over time with resolution of the empyema itself [11–13].

Thus, current practices of open chest drainage for patients with empyema are best described as an OWT including Clagett windows, MEFs, and EFs. The aim of our study was to review our own practice and compare short and long-term outcomes of OWT for management of empyema, in the era of minimally-invasive surgery, endobronchial valves (EBVs), and fibrinolytic therapy.

## Methods

We identified all patients who underwent OWT at our institution between 2010 and 2020 by querying International Classification of Disease (ICD)-9 code 510 (empyema), and ICD-10 J86.0 (pyothorax with fistula). These ICD codes was then cross-matched to relevant current procedural terminology (CPT) codes including: 32035 (thoracostomy with rib resection), 23,036 (thoracostomy with flap drainage), 32,220 (release of lung), 32,225 (partial release of lung), 32,810 (closure chest after drainage), 32,905 and 32,906 (both revise and repair chest wall). These procedure codes were also cross-referenced to ICD-9 and ICD-10 procedure codes in order to capture additional patients.

A retrospective review of all patients undergoing any of the three OWT procedures was performed under an institution-approved IRB protocol. Statistical analysis included modeling survival data using a standard Kaplan Meier curve. Additionally, 10-year survival estimates at the time of the index operation was calculated for each patient using the Charlson Comorbidity Index (CCI) score; points were given for age at time of surgery (age 50–50, 60–69, 70–79 and ≥ 80 given + 1, 2, 3, and 4 points respectively), history of myocardial infarction, congestive heart failure, peripheral vascular disease, cerebral vascular accident or transient ischemic attack, dementia, chronic obstructive pulmonary disease, connective tissue disease, peptic ulcer disease (all + 1 point), liver disease (+ 1 mild = chronic hepatitis or cirrhosis without portal hypertension; + 2 moderate to severe = cirrhosis and portal hypertension without or with variceal bleeding history), diabetes mellitus (none/diet-controlled + 0, uncomplicated + 1, end-organ damage + 2), hemiplegia (+ 2), moderate to severe chronic kidney disease (Cr > 3 mg/dL or status post kidney transplant + 2), solid tumor (localized + 2, metastatic + 6), leukemia (+ 2), lymphoma (+ 2), acquired immunodeficiency syndrome (+ 6).

## Results

### Demographics

We identified 18 patients who had undergone an OWT. Their demographics are outlined in Table 1. Patients ranged in age from 31 to 79. Nine patients (50%) had a history significant for smoking. All but 3 patients had significant pre-existing comorbidities as graded by the CCI, an estimate of 10-year survival based on individual comorbidities at the time of the index operation. Two of the 3 patients without pre-existing comorbidities as measured by the CCI had a post-traumatic cause of empyema (one occurring in an otherwise healthy 47-year-old male after a gun-shot wound to the chest and the other in a 31-year-old male with cerebral palsy after a gastrostomy-tube was inadvertently tunneled into the pleural space). Of the patients without comorbidities tabulated by CCI, 2 of 3 did not require readmission after their index operation other than for scheduled delayed closure, and they are both still living. The third patient with underlying cerebral palsy died secondary to complications. The most common comorbidities among our patient population were metastatic cancer (*n* = 7) and hypertension/coronary artery disease, which is not factored into CCI (*n* = 7). Another common comorbidity was COPD or reactive airway disease (*n* = 4).

**Table 1:** Demographic characteristics of OWT cohort

Cohort Demographics, ***N*** = 18	
Patient age at procedure (years), M ± SD Patient age range	59.44 ± 14.67 31–79
Gender, *n (%)*	
Male	11 (61.1)
Female	7 (38.9)
Ethnicity/Race, *n* (%)	
Non-Hispanic, Caucasian	9 (50.0)
Black, African American	4 (22.2)
Asian	1 (5.6)
Hispanic	1 (5.6)
Other, Unknown	3 (16.6)
Smoking History, *n* (%)	
Yes	9 (50.0)
No	9 (50.0)
Comorbidities, *n*	
Cancer, metastatic	7
Cancer, local	2
COPD or Reactive airway disease	4
HTN/CAD	7
Organ transplant	1
HIV/AIDs	1
CVA/stroke	2
Cerebral Palsy	1
None	2
Charlson Comorbidity Index Score	Predicted 10-year survival
0 (*n* = 3)	98%
1 (*n* = 1)	96%
3 (*n* = 2)	77%
5 (*n* = 3)	21%
6 (*n* = 4)	2%
8 (*n* = 1)	0%
9 (*n* = 3)	0%
12 (*n* = 1)	0%
Indication for OWT*, n (%)*	
Post-resectional BPF	9 (50.0)
Post-pneumonectomy	5
Post-lobectomy	4
Parapneumonic Empyema	5 (27.8)
Esophageal Related	1 (5.6)
Subdiaphragmatic Abscess	1 (5.6)
Post-traumatic BPF	2 (11.1)

Abbreviations: *AIDS* acquired immunodeficiency syndrome, *BPF* bronchopleural fistula, *CAD* coronary artery disease, *COPD* chronic obstructive pulmonary disease, *CVA* cerebrovascular accident, *HIV* human immunodeficiency virus, *HTN* hypertension, *M* mean, *OWT* open window thoracotomy, *SD* standard deviation

### Operative details

Operative details of the procedures are listed in Table 2. The most common indication for OWT was a post-resectional BPF (50% with *n* = 4 post-lobectomy and *n* = 5 post-pneumonectomy). In the majority of post-resectional BPF patient population (*n* = 6), presence of empyema meant less invasive surgical options for stump closure such as stump revision or filling post-resectional dead space with muscle or omentum given extent of contamination were not feasible. In one patient who suffered from a bronchial stump fistula after a right upper lobectomy, however, an endobronchial stent was trialed to protect the remaining lung from spillage while allowing time for the BPF to heal. Given a persistent loculated apical cavity in the setting of mycobacterium avium-intracellulare infection, an OWT was ultimately performed.

**Table 2:** Short and long-term outcomes of OWT cohort

Cohort Outcomes, ***N*** = 18	
Patients failed less invasive interventions	11
VATS decortication only	4
Failed VATS w/ conversion to open decortication	1
VATS w/ later decortications ×2 and Amplatz	1
Open decortication only	3
Minimally invasive intervention only	2 (*n* = 1 EBV, *n* = 1 endobronchial stent)
Ribs resected (number), M ± SD; range	2.5 ± 1.2; 1–6
NPWT utilized, *n (%)*	9 (50.0)
Kerlix packing, *n (%)*	9 (50.0)
Delayed Closure, *n (%)*	
Yes	11 (61.1)
Latissimus dorsi pedicled flap	6^a^
Pectoralis major pedicled flap	1
Free flap	2
Re-approximation of surrounding tissue	2
No, due to death	6 (33.3)
Unknown, lost to follow up	1 (5.6)
Time from index surgery to delayed closure, M ± SD (months); range	4.8 ± 6.7; 3 days – 22.2 months
Patient Deceased, n (%)	
Yes, total	12 (66.6)
Yes, within 30 days post-operatively	3
-*N* = 2 Respiratory failure, sepsis	
-*N* = 1 Acute myocardial infarction	
Yes, within 90 days post-operatively	1
-Tracheoinnominate fistula, hemorrhage, sepsis	
Yes, > 90 days post-op	8
No	6 (33.3)
Major post-operative complication (within 90 days), *n(%)*	
Recurrent infection after closure	1 (5.6)
DVT	1 (5.6)
Pulmonary Embolism	1 (5.6)
Stroke	1 (5.6)
MI	1 (5.6)
OR take-back	2 (11.1)
-POD1: Hemorrhage	
-POD 7: Subcutaneous emphysema	
Readmission	
Yes, total	7 (38.9)
Yes, within 30 days	3
-*N* = 1 scheduled closure	
-*N* = 1 fall, altered mental status	
-*N* = 1 dyspnea	
Yes, within 90 days	4
-*N* = 2 scheduled closure	
-*N* = 1 aspiration pneumonia	
-*N* = 1 bleeding from OWT	
No	6 (33.3)
Not applicable, death on primary admission	5 (27.8)
Overall Survival (months), M ± SD	24.0 ± 32.2

^a^1 patient re-opened for recurrent infection and repeat OWT/Eloesser flaps

Abbreviations: *DVT* deep vein thrombosis, *EBV* endobronchial valve, *MI* myocardial infarction, *NPWT* negative pressure wound therapy, *VATS* video-assisted thoracoscopic surgery

Less invasive methods (Table 2) were trialed in *n* = 11 patients prior to OWT. These patients included 5 with parapneumonic empyema, 3 with post-resectional BPF, 2 with post-traumatic BPF, and 1 with subdiaphragmatic abscess. None of the patients were deemed appropriate for fibrinolytic therapy. Four patients underwent unsuccessful VATS prior to OWT. One patient underwent an initially unsuccessful VATS with the surgeon converting to open decortication. Another patient underwent an unsuccessful VATS, two later unsuccessful open decortications and also failed an Amplatz VSD device for BPF closure. Three patients failed open decortication prior to their OWT, and *n* = 2 patients failed minimally invasive options with one failing an EBV and another failing an endobronchial stent.

Mean number of ribs resected were 2.5 with a range notable for 1–6 ribs. The one patient who had 6 ribs resected suffered from cerebral palsy and had a traumatic gastro-pleural fistula secondary to a mispositioned percutaneous gastrostomy tube at an outside institution. He underwent multiple attempts to repair the fistula, debridements of the chest wall, and persistent empyema complicated by Klebsiella sepsis at the time of his transfer. Part of his lower lobe had been resected with complete loss of left hemi-diaphragm and chest wall domain. He ended up requiring removal of ribs 6–11 to collapse the chest and provide definitive obliteration of the empyema cavity. Simultaneously, a new G-tube was placed with an intercostalis muscle flap to buttress the space between the stomach still plastered onto the chest wall and the pericardium. Unfortunately, the patient died 43 days after his OWT due to tracheoinnominate fistula.

NPWT was utilized for 50% of patients, with Kerlix packing used in patients who did not undergo NPWT. NPWT was primarily used for patients who only had trapped lung, and had no underlying airleak as that would not allow the negative-pressure device to hold suction. The was no trend for NPWT vs Kerlix over time, and 6 of 9 NPWT therapy patients were successfully closed by the end of the study. One patient was lost to follow up and two were deceased prior to closure. Eleven of 18 patients (61.1%) underwent delayed closure, with the most common means of closure being a latissimus dorsi pedicled flap (6 of 11 closed patients). The mean time to flap closure was 4.8 months with a range of 3 days – 22.2 months. Six patients were not closed as they died in the interim and in one patient, closure status is unknown as they were lost to follow up. In 1 patient who was closed 109 days after the index operation, BPF with empyema recurred within 2 months of closure and was managed with repeat thoracotomy and MEF creation. During the closure there were no signs of ongoing infection; though there was some fibrinous material debrided.

### Outcomes

**Fig. 2:**
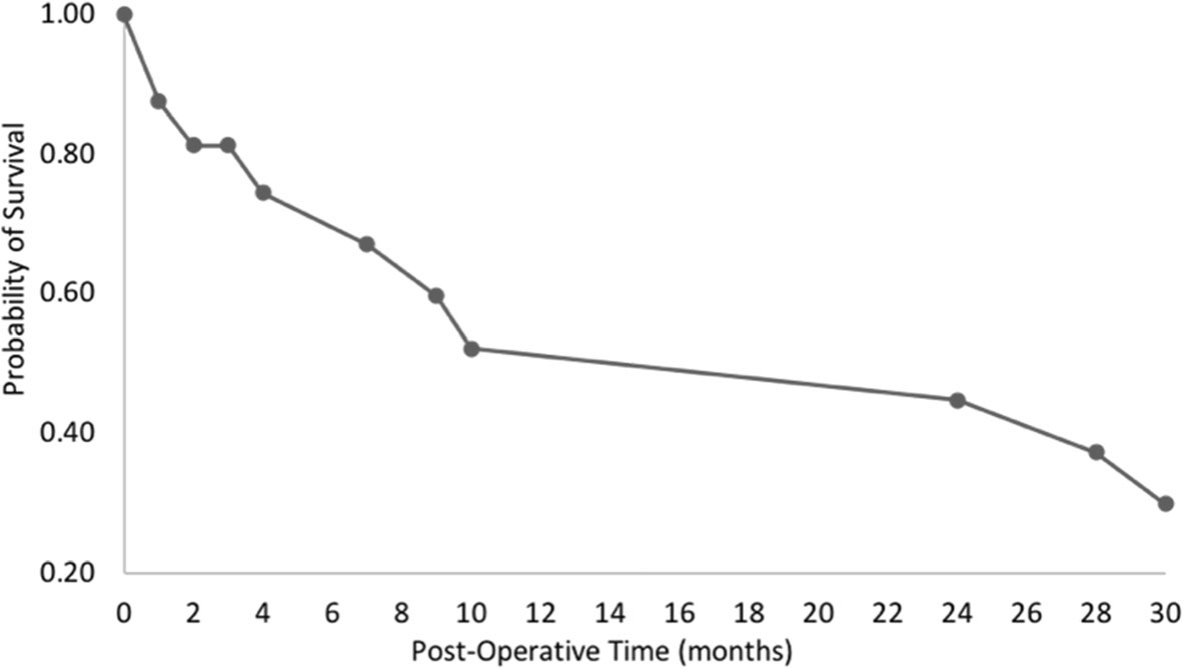
Kaplan Meier curve modeling survival probability for OWT cohort

At the end of the review period, 12 patients (66.6%) were deceased, with 3 (16.7%) patients dying within 30 days postoperatively with the following etiologies: two patients with respiratory failure/sepsis and one with acute myocardial infarction. A significant long-term complication was recurrent infection after closure (1 or 5.6% of patients). Additionally, 2 patients required OR takeback within 30 days, one for hemorrhage and the other for subcutaneous emphysema resulting in respiratory failure requiring intubation and chest tube placement in the contralateral pleural space to the OWT site. Total readmissions within 90 days included 7 patients (38.9%); though 5 patients (27.8%) had died on admission for index surgery. The mean overall survival was 24 months. Our Kaplan Meier curve shows the probability of survival after OWT to 1 month is 88 and 30% at 30 months (Fig. 2).

In our study, most patients who underwent OWT were debilitated at baseline – i.e. in patients who underwent initial lung resection, it appears that 5 of them were poor surgical candidates to begin with given frailty and metastatic disease burden, irrespective of their underlying severe pleural space infection as evidenced by their high CCI Scores (*n* = 13 with a score ≥ 5 indicating 21% 10-year survival or less) (Table 1). Two of these patients nonetheless underwent resection despite their metastatic cancer given their young ages, 37 and 46. Interestingly, only 2 patients in our cohort elected for hospice in the year after OWT and 1 patient was compassionately extubated before OWT closure in the setting of respiratory failure secondary to his underlying pneumonia. It is unknown if hospice options were discussed with all these patients, but in retrospect it is unclear if all the patients in this cohort should have been offered major lung resection to begin with.

## Discussion

No literature has been published in the last 10 years looking at outcomes of OWT. Our study illustrates the often-significant comorbidities of patients undergoing OWT in the modern era as well as the associated poor outcomes for patients and pitfalls that continue to be associated with this morbid procedure. There were two previous major studies published on this subject prior to the era of widespread use of fibrinolytic therapy. The first is a study by Thourani et al. in 2003 which examined 78 patients from 1975 to 2001 who received strictly MEFs for empyema thoracis [14]. The second is a study by Reyes et al. in 2010 which looked at 78 patients who underwent OWT from 1998 to 2008 [15]. However, our cohort is distinct from both of theirs for multiple reasons. Compared to Reyes’ study which found a 6% 30-day mortality and Thourani’s study which found a 5% 30-day mortality, our study demonstrates a 12% 30-day mortality predicted by a Kaplan-Meyer curve or 16.7% based on raw data (*n* = 3 of 18 deceased at 30 days, Fig. 2).

Our increased mortality and smaller cohort are in part explained by the advent of EBVs and fibrinolytics, and improved conservative interventions for primary BPF and empyema management. Given these advances, fewer patients go to the operating room for OWT as their index case, and often those who do undergo OWT have either severe disease processes making conservative therapy not possible – such as the many patients with metastatic cancer in our cohort (*n* = 7) who were not candidates for lytic therapy, or they failed less-invasive surgical interventions, which was the case for 11 of our patients. Less-invasive methods tended to fail in patients with a chronic empyema cavity or in patients where too much bleeding was encountered with typical decortication. Furthermore, in 2 non-cancer parapneumonic empyema patients, lytic therapy was considered but the risk of bleeding was deemed too high - in one case in the setting of heparin induced thrombocytopenia and in the other case the patient’s initial presentation included massive hematemesis from a bleeding esophageal ulcer while on anti-coagulation for recent stroke in addition to necrotizing pneumonia with parapneumonic empyema.

Additionally, in current practice decortication with same day closure (either VATS or via open thoracotomy) are considered the procedures of choice before OWT as they are known to have superior outcomes. This was unsuccessfully attempted in 11 patients in our cohort who ultimately required OWT [16, 17]. Moreover in both Reyes’ and Thourani’s study the primary indication for OWT was for parapneumonic empyema, whereas in our study it was postoperative BPF with associated empyema and advanced cancer. Fibrinolytic therapy is therefore challenging in this patient population given risk of contaminating contralateral healthy lung given patent fistula and bleeding given metastatic deposits in the pleural space [1, 14, 18]. The wide-use of EBVs has also changed the management of persistent air leaks, albeit only in sterile fields; this was trialed and failed in one of our patients [19].

Furthermore, our study supports the recent work of Nayak et al. 2020, which analyzed the epidemiology and trends in management of thoracic empyema from 1996 to 2015 [20]. Like our study, they used the CCI to analyze morbidity and mortality risk in their population. They observed an increased incidence over time of thoracic empyema in patients aged 50–70 and postulated that this trend both reflects a change in the etiology of empyema from risk factors affecting a younger or more at risk population (intravenous drug use, tuberculosis) as well as the greater presence of risk factors such as COPD and diabetes – both independent risk factors for empyema development – in the aging population [21]. Our study likewise had older patients (an average age of 59.44) with significant comorbidities as measured according to the CCI, supporting the changing epidemiological trends reported in Nayak et al. [20]

There are several limitations to our study. First, the cohort of OWT patients (*n* = 18) in this study is limited by its small size. However, it still provides a valuable illustration of the various etiologies for which OWT is still indicated, most notably for trapped lung that cannot be expanded via VATS or open decortication or post-resectional dead space which is a nidus for infection in the setting of BPF. Additionally, our study further illustrates – similarly to past studies [9, 10, 22] -- that NPWT can be utilized as potential way to obliterate the pleural space and manage an open chest in absence of an airleak, as this method ultimately led to successful closure in 6 of 9 patients with NPWT.

Finally, a particularly undesirable, yet possible outcome of empyema management with OWT is premature window closure leading to recurrent infection, as seen in one patient in our cohort (Table 2). This patient was significantly immunocompromised at the time of closure given his underlying stage IV non-small cell lung cancer, which likely increased his risk of recurrent infection. Cases such as this illustrates the need to be wary of the possibility of recurrent or ongoing sub-clinical infection in patients who seem otherwise well and ready for closure. This is particularly poignant for immunocompromised patients who may not mount a clinical, symptomatic response (fever, leukocytosis) to ongoing infection. While there is no consensus as to when to close OWT patients, particular care should be taken in the immunocompromised patient to give enough time to truly decontaminate the space and ensure that colonization of the pleural space has decreased to < 10^5^ colonies/hpf.

## Conclusions

Patients that undergo OWT are a sick population at baseline many of whom are not candidates for fibrinolytic therapy and EBVs given their underlying cancer, risk of bleeding, and/or BPF in the setting of contaminated pleural space. The poor outcomes associated with OWT in current practice are unsurprising as patients receiving this operation are either too sick for other interventions or are out of other treatment options. A pitfall to avoid in management of OWT patients is early closure of the window to avoid the dire effects of reinfection. Further studies are needed to compare fibrinolytics versus all surgical interventions for empyema, albeit the results can be expected to be better for the former cohort of patients.

## Data Availability

The datasets used and/or analyzed during the current study are available from the corresponding author on reasonable request.

## Abbreviations

VATS: Video assisted thoracoscopic surgery
OWT: Open window thoracostomy
EF: Eloesser flap
MEF: Modified Eloesser flap
NPWT: Negative pressure wound therapy
CCI: Charlson Comorbidity Index
BPF: Bronchopleural fistula
